# Pulsed Electromagnetic Field (PEMF) Treatment Ameliorates Murine Model of Collagen-Induced Arthritis

**DOI:** 10.3390/ijms24021137

**Published:** 2023-01-06

**Authors:** Ju-Eun Hong, Chang-Gun Lee, Soonjae Hwang, Junyoung Kim, Minjeong Jo, Da-Hye Kang, Sang-Hyeon Yoo, Woo-Seung Kim, Yongheum Lee, Ki-Jong Rhee

**Affiliations:** 1 Department of Biomedical Laboratory Science, College of Software and Digital Healthcare Convergence, Yonsei University MIRAE Campus, Wonju 26493, Republic of Korea; 2 Department of Medical Genetics, Ajou University School of Medicine, Suwon 16499, Republic of Korea; 3 Department of Biochemistry, Lee Gil Ya Cancer and Diabetes Institute, GAIHST, Gachon University College of Medicine, Incheon 21999, Republic of Korea; 4 Department of Biomedical Engineering, College of Software and Digital Healthcare Convergence, Yonsei University MIRAE Campus, Wonju 26493, Republic of Korea; 5 Department of Molecular Cell Biology, Sungkyunkwan University School of Medicine, Suwon 16419, Republic of Korea

**Keywords:** pulsed electromagnetic field, rheumatoid arthritis, collagen-induced arthritis

## Abstract

Rheumatoid arthritis (RA) is an autoimmune disease of the joint synovial membranes. RA is difficult to prevent or treat; however, blocking proinflammatory cytokines is a general therapeutic strategy. Pulsed electromagnetic field (PEMF) is reported to alleviate RA’s inflammatory response and is being studied as a non-invasive physical therapy. In this current study, PEMF decreased paw inflammation in a collagen-induced arthritis (CIA) murine model. PEMF treatment at 10 Hz was more effective in ameliorating arthritis than at 75 Hz. In the PEMF-treated CIA group, the gross inflammation score and cartilage destruction were lower than in the untreated CIA group. The CIA group treated with PEMF also showed lower serum levels of IL-1β but not IL-6, IL-17, or TNF-α. Serum levels of total anti-type II collagen IgG and IgG subclasses (IgG1, IgG2a, and IgG2b) remained unchanged. In contrast, tissue protein levels of IL-1β, IL-6, TNF-α, receptor activator of nuclear factor kappa-Β (RANK), RANK ligand (RANKL), IL-6 receptor (IL-6R), and TNF-α receptor1 (TNFR1) were all lower in the ankle joints of the PEMF-treated CIA group compared with the CIA group. The results of this study suggest that PEMF treatment can preserve joint morphology cartilage and delay the occurrence of CIA. PEMF has potential as an effective adjuvant therapy that can suppress the progression of RA.

## 1. Introduction

Rheumatoid arthritis (RA) is a systemic, chronic, inflammatory disease characterized by the accumulation and persistence of inflammatory infiltrates in the synovial membrane that leads to synovitis and the destruction of the joint architecture and results in impaired function [1,2]. Uncontrolled inflammation of the synovial tissue subsequently results in the destruction of surrounding cartilage and bone, which causes joint deformation [3]. Patients who are refractive to treatment develop severe joint dyskinesia within 10 to 20 years, and it has been reported that their average life expectancy is shorter than that of the general population [4]. Bone destruction occurs in more than 70% of RA patients within 2 years of disease onset, and joint damage is known to progress more rapidly in the first year [5]. The degree of joint destruction is correlated with the severity of inflammation. Although the exact cause of RA remains unclear, a genetic predisposition, environmental factors, and immunological factors are thought to interact in a complex manner to cause arthritis [6]. Controlling inflammation, preventing/delaying joint damage, relieving pain, and maintaining joint function are ultimate goals to maintain the patient’s quality of life [7]. For RA treatment, a pyramidal therapeutic approach begins with the administration of anti-rheumatic drugs and eventually stronger immunosuppressants [8]. However, this approach has limitations and does not completely prevent joint damage. Furthermore, pharmacologic agents that are commonly used during RA treatment are often costly and possess numerous side effects [9]. Therefore, it is important to focus on the early stages of the disease to intervene in pre-arthritis stages and delay the progression of chronic RA [10].

Electromagnetic fields (EMFs) have been used therapeutically in recent decades for a wide range of ailments [11]. Among different EMF techniques, pulsed electromagnetic field (PEMF) produces a low-frequency magnetic field with specific waveforms and amplitudes that range between 6 and 500 Hz and are released in very short, pulsed intervals [12]. Therapies using PEMF do not require direct contact with the target tissue; thus, they are non-invasive. Moreover, it has the added benefit of penetrating deep tissues. Optimizing the physical signal characteristics (frequency, intensity, duration, and waveform) of PEMF to the desired therapy has led to beneficial results [13]. Recent advances in alternative and complementary medicine have led to an increasing interest in the therapeutic use of PEMF [13,14]. The effectiveness of PEMF has been reported regarding the management of therapeutically recalcitrant diseases of the musculoskeletal system [15]. In another study, RA patients were treated with PEMF for 30 min, and the pain level was assessed using the McGill Pain Questionnaire [16]. The PEMF treated patients reported a decrease in pain compared with the non-treated patients. However, this brief PEMF treatment is unlikely to have exerted a lasting effect on the arthritic tissue. In a murine model of osteoarthritis, PEMF treatment ameliorated joint pain and arthritis by reducing the proinflammatory cytokines TNF-α and IL-6 [17]. These reports suggest that the pathologic cellular immune response in arthritic joints can be significantly suppressed with the use of PEMF.

Collagen-induced arthritis (CIA) murine models have been used to understand human RA because CIA-induced mice show similar patterns of synovitis, pannus formation, erosion of cartilage and bone, fibrosis, and loss of joint mobility [18]. Clinical signs in CIA mice typically develop 21–25 days after the initial injection of collagen and manifest as polyarthritis, which peaks in severity at approximately day 35 post-injection [19]. The CIA model is also characterized by the generation of autoantibodies predominated by anti-collagen IgG2 subclasses which activate immune cells and the secretion of TNF-α, IL-6, and IL-1β [20]. The detection of proinflammatory cytokines in the joint tissues, synovial fluid, and serum of RA patients suggests that these cytokines play an important role in local/general inflammatory responses [21]. These inflammatory cytokines cooperate with the receptor activator of nuclear factor kappa B ligand (RANKL) to increase the formation of osteoclasts, which results in bone and joint destruction [22]. An extremely important feature of CIA that makes it a valid model for RA is the expression of proinflammatory cytokines, including TNF-α and IL-1β, in the joints of mice with arthritis. The blockade of these molecules results in the reduced clinical and histological severity of disease [23].

In the current study, we have investigated the anti-inflammatory effects of PEMF in a CIA murine model. We meticulously examined histological damage, the production of inflammatory cytokines, and autoantibody formation. We found that PEMF treatment delayed the progression of RA, reduced joint tissue damage, and attenuated inflammatory mediators. Our results suggest that PEMF treatment can be used as an alternative/complementary therapy to conventional treatment modalities for RA.

## 2. Results

### 2.1. PEMF Ameliorates CIA in Mice

**Figure 1:**
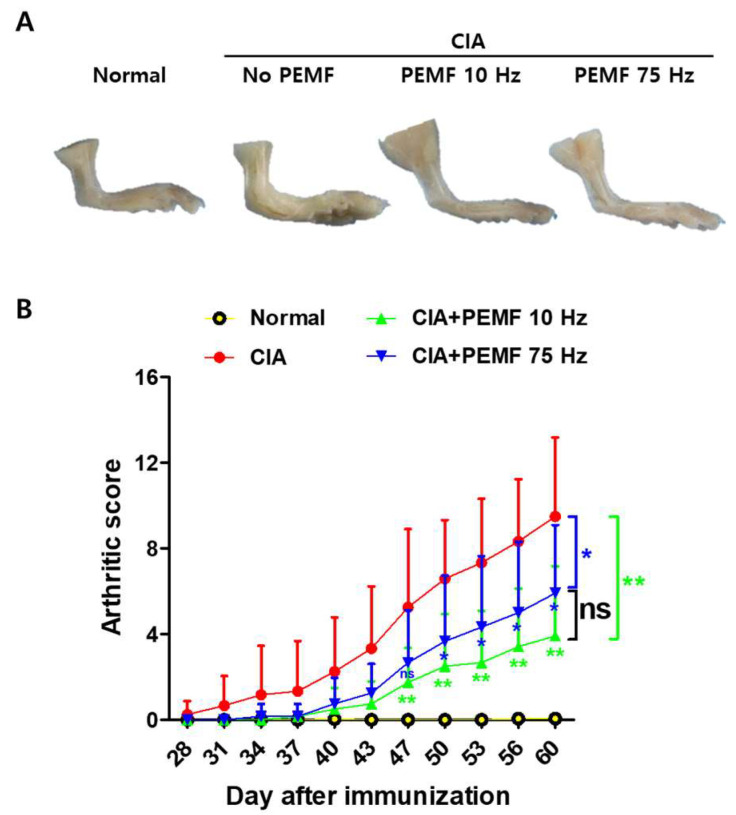
Effects of PEMF on CIA progression in mice. The severity of arthritis in CIA mice was evaluated by determining the clinical arthritis score beginning from 7 days after the second immunization (i.e., day 28). (**A**) Macroscopic appearance of the ankle joint. (**B**) Arthritic score. Grouped quantitative data of arthritic scores are presented as mean ± SD. Normal, *n* = 12; CIA, *n* = 12; CIA+PEMF 10 Hz, *n* = 12; CIA+PEMF 75 Hz, *n* = 12. Data were pooled from two independent experiments. Significance was measured using a two-tailed Student’s *t*-test. Significance levels were * *p* < 0.05 and ** *p* < 0.01, while ns means the results were not significant.

To examine whether PEMF treatment attenuates CIA in mice, DBA/1 mice were immunized twice with bovine type II collagen (days 0 and 21), and PEMF treatment was initiated 7 days after the second immunization, as detailed in Materials and Methods. Antecedent studies suggested that PEMF pulse frequency effects on cells and tissues associated with RA range from 10 to 75 Hz [24]. Therefore, the pulse frequencies used in the experiment were set at 10 Hz (145 Gauss) or 75 Hz (85.9 Gauss), and the intensity was a mono-phasic stimulation of 15 mT (pulse duty ratio: 30%) [25]. The arthritic score for the CIA control group without PEMF treatment gradually increased after day 28 and reached 9.5 ± 1.06 by day 60 (Figure 1). Arthritic scores for both CIA+PEMF groups showed a delay, but they gradually increased by day 60. At day 47, the CIA+PEMF groups began to show statistically significantly lower arthritic scores compared with the CIA group. This difference gradually increased, and by day 60, the arthritis scores of CIA+PEMF 10 Hz and CIA+PEMF 75 Hz were 3.91 ± 0.94 and 5.92 ± 0.92, respectively. The arthritic score modestly decreased in the PEMF 10 Hz treated mice compared with the PEMF 75 Hz mice, but it did not reach statistical significance. These results suggest that PEMF has ameliorative effects on CIA progression.

### 2.2. PEMF Reduces Articular Inflammation and Erosion

**Figure 2:**
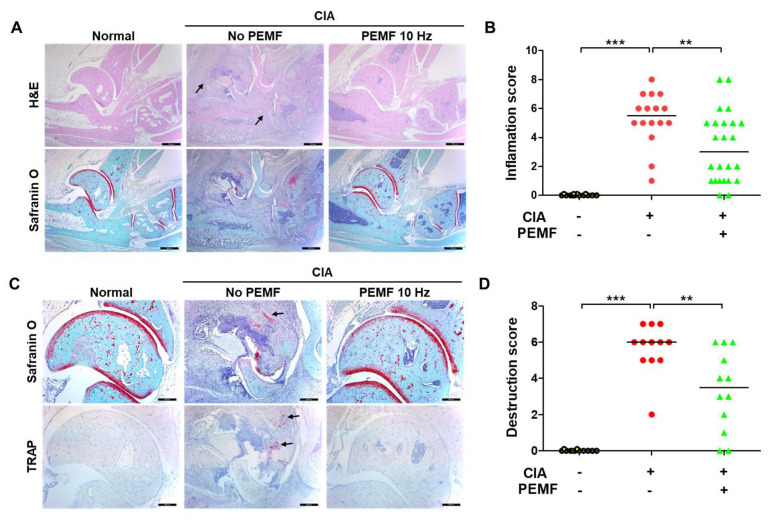
Effects of PEMF (10 Hz) on joint inflammation and cartilage destruction. (**A**) H&E (upper panel) and safranin O (lower panel) stained images of hind paw tissues. The magnification is ×40. Bar, 500 μm. Inflammatory cell infiltration is indicated by black arrows. (**B**) Inflammation scoring of H&E stained tissue. (**C**) Representative safranin O (upper panel) and tartrate-resistant acid phosphatase (TRAP; lower panel) stained images of hind paw tissues. The magnification is ×100. Bar, 200 μm. Cartilage destruction is indicated by black arrows. (**D**) Histopathological scoring of safranin O stained tissue based on the destruction of cartilage and bone. Significance was measured using a two-tailed Student’s *t*-test (** *p* < 0.01 and *** *p* < 0.001). Bars indicate the median. Each dot represents one mouse.

The severity of arthritis in CIA mice was evaluated using H&E staining of the histological sections of hind paws. Mice in the CIA group showed severe histopathological changes, including massive infiltration of inflammatory cells, pannus formation, and the erosion of bone and cartilage, whereas mice in the CIA+PEMF group showed a marked reduction in inflammatory cell infiltration and erosion of bone and cartilage tissues (Figure 2A). Consistent with the H&E staining results, inflammation scores significantly decreased in CIA+PEMF mice compared with the CIA mice (Figure 2B). In addition, CIA+PEMF mice showed a significant decrease in tartrate-resistant acid phosphatase (TRAP)-positive osteoclasts in the bone erosion region compared with CIA mice (Figure 2C). The reduction in cartilage damage was further confirmed by quantifying cartilage destruction using safranin O staining (Figure 2D). Based on these histological results, PEMF treatment exhibited an ameliorative effect on CIA progression by reducing pannus, inflammation, and cartilage erosion in the ankle joint.

### 2.3. PEMF Does Not Decrease Serum Proinflammatory Cytokines

**Figure 3:**
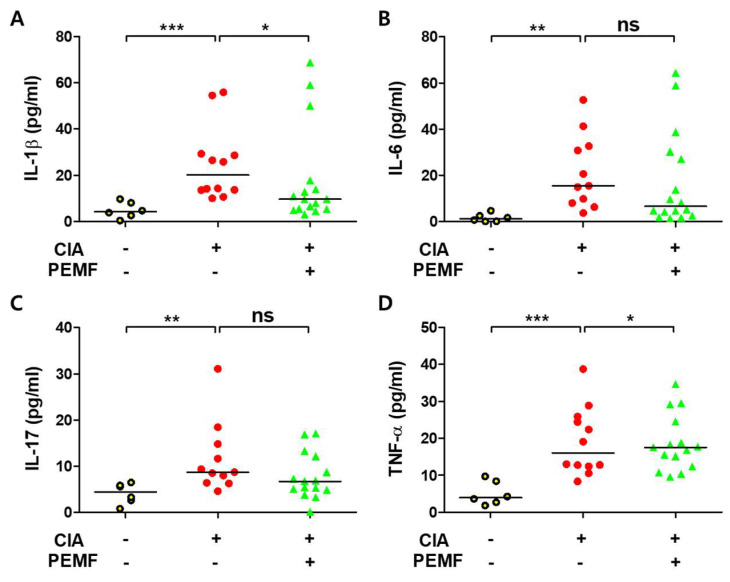
Effects of PEMF (10 Hz) on serum proinflammatory cytokines. (**A**) IL-1β, (**B**) IL-6, (**C**) IL-17, and (**D**) TNF-α were measured by ELISA in serum obtained on day 60. Grouped quantitative data are presented as medians. Significance was measured using a two-tailed Student’s *t*-test (* *p* < 0.05, ** *p* < 0.01, and *** *p* < 0.001; ns, not significant). Bars indicate the median. Each dot represents one mouse.

Levels of serum proinflammatory cytokines (IL-1β, IL-6, IL-17, and TNF-α) were assessed at day 60 by ELISA. As expected, the levels of proinflammatory cytokines in the sera of CIA mice were significantly higher than those of normal mice (Figure 3). The level of serum IL-1β significantly decreased in the CIA+PEMF group compared with the CIA group (Figure 3A). Serum IL-6 and IL-17 levels were also lower in the CIA+PEMF group, but they did not reach statistical significance (Figure 3B,C). Contrary to expectations, the serum levels of TNF-α did not decrease in the CIA+PEMF group, but they statistically significantly increased compared with the CIA group (Figure 3D). These findings suggest that PEMF treatment does not decrease serum proinflammatory cytokines.

### 2.4. PEMF Does Not Inhibit Autoantibody Formation in CIA Mice

**Figure 4:**
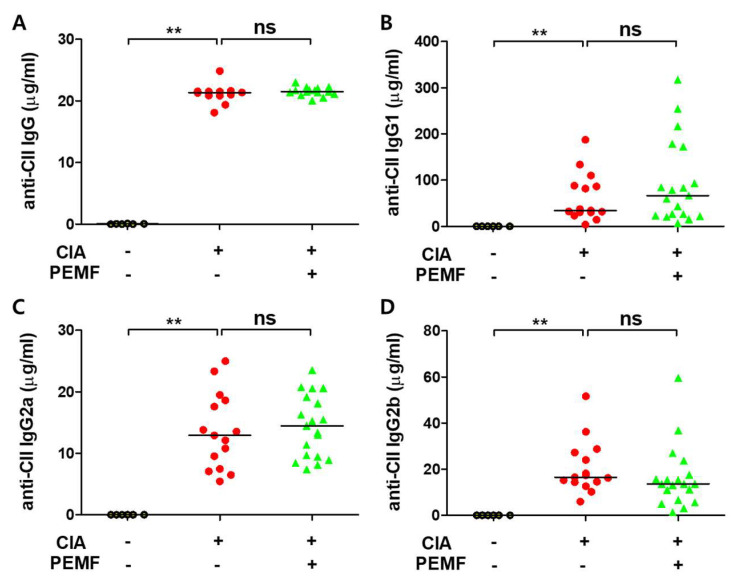
Effects of PEMF (10 Hz) on serum anti-type II collagen autoantibody levels. Levels of (**A**) anti-type II collagen IgG and its subtypes, including (**B**) IgG1, (**C**) IgG2a, and (**D**) IgG2b, were measured by ELISA in serum obtained on day 60 from each mice group. Grouped quantitative data are presented as the median. Significance was measured using a two-tailed Student’s *t*-test (** *p* < 0.01 and ns, not significant). Bars indicate the median. Each dot represents one mouse.

Anti-collagen autoantibodies, especially IgG2a and IgG2b isotypes, play a critical role in the development of RA [26]. Since PEMF ameliorated pathologic destruction in CIA mice, the effect of PEMF on the formation of collagen-specific autoantibodies was examined by ELISA. As expected, the levels of total anti-type II collagen-specific IgG antibody and the levels of anti-type II collagen-specific IgG1, IgG2a, and IgG2b were all elevated in CIA mice (Figure 4). However, levels of total anti-type II collagen-specific IgG and IgG subclasses were similar between the CIA and CIA+PEMF groups (Figure 4). Serum anti-type II collagen-specific IgG2b levels modestly decreased in the CIA+PEMF group compared with the CIA group, but they did not reach statistical significance (Figure 4D). These results suggest that PEMF treatment does not inhibit the formation of autoantibodies in CIA mice.

### 2.5. PEMF Decreases Proinflammatory Cytokines in Ankle Joint Tissues

**Figure 5:**
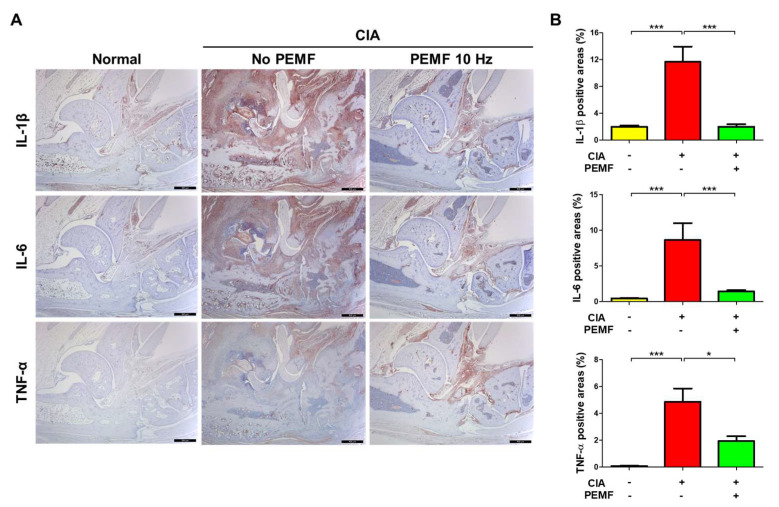
Effects of PEMF (10 Hz) on IL-1β, IL-6, and TNF-α expression in ankle joint tissues. (**A**) Immunohistochemical staining of IL-1β, IL-6, and TNF-α in ankle joint paraffin sections. The magnification is ×40. Bar, 500 μm. (**B**) Histograms showing the percentage of positive tissue area for IL-1β, IL-6, and TNF-α. Data are presented as the mean ± standard error of the mean (SEM). Normal, *n* = 7; CIA, *n* = 6; CIA+PEMF 10 Hz, *n* = 10. Significance was measured using a two-tailed Student’s *t*-test (* *p* < 0.05 and *** *p* < 0.001).

In RA and CIA, the increased production of various proinflammatory cytokines, including IL-1β, IL-6, and TNF-α, leads to joint inflammation and bone erosion [27]. However, we only observed a decrease in serum IL-1β levels but not serum IL-6 and TNF-α in the CIA+PEMF group compared with the CIA group (Figure 3). Therefore, we examined the levels of these proinflammatory cytokines in ankle joint tissues by immunohistochemical analysis. Consistent with the serum ELISA results, we found significantly lower tissue IL-1β in the CIA+PEMF group compared with the CIA group (Figure 5A). In contrast to serum IL-6 and serum TNF-α levels, tissue IL-6 and serum TNF-α levels were significantly lower in the CIA+PEMF group compared with the CIA group (Figure 5B,C). These results suggest that PEMF reduced the levels of proinflammatory cytokines in the ankle joint tissue.

### 2.6. PEMF Reduces RANKL and RANK Levels in Ankle Joint Tissues

**Figure 6:**
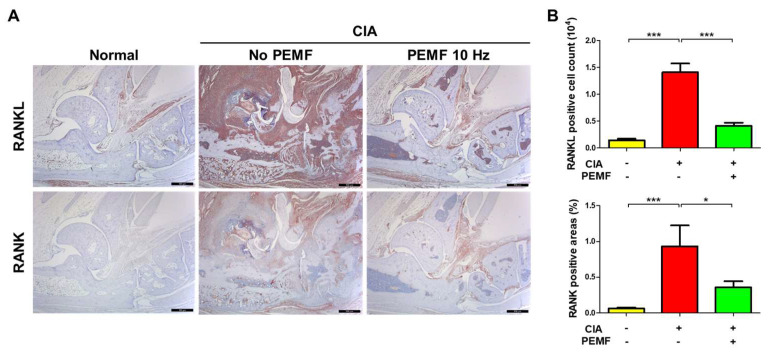
Effects of PEMF (10 Hz) on RANKL and RANK levels in ankle joint tissues. (**A**) Immunohistochemical staining of RANKL and RANK in ankle joint paraffin sections. The magnification is ×40. Bar, 500 μm. (**B**) The percentage of RANKL-positive cells and RANK-positive tissue. Data are presented as the mean ± SEM. Normal, *n* = 7; CIA, *n* = 6; CIA+PEMF 10 Hz, *n* = 10. Significance was measured using a two-tailed Student’s *t*-test (* *p* < 0.05 and *** *p* < 0.001).

RANKL-RANK signaling is known to be a potent cause of osteoclast differentiation, which is involved RA-mediated bone destruction [27,28]. Therefore, we examined the levels of RANK and RANKL in ankle joint tissue by immunohistochemical analysis. CIA mice showed a significant increase in RANKL and RANK levels in ankle joint tissues, whereas the tissues levels of RANKL and RANK decreased significantly in CIA+PEMF mice compared with CIA mice (Figure 6). These results suggest that RANKL-RANK signaling was ameliorated by PEMF treatment, which thereby reduced the number of activated osteoclasts and lowered bone and cartilage erosion in ankle joint tissues.

### 2.7. PEMF Reduces IL-6R and TNFR1 Levels in Ankle Joint Tissues

**Figure 7:**
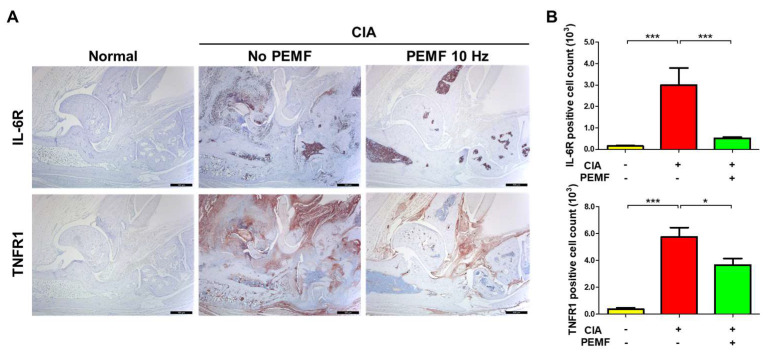
Effects of PEMF (10 Hz) on IL-6R and TNFR1 levels in ankle joint tissues. (**A**) Immunohistochemical staining of IL-6R and TNFR1 in ankle joint paraffin sections. The magnification is ×40. Bar, 500 μm. (**B**) The percentage of IL-6R-positive and TNFR1-positive cells. Data are presented as the mean ± SEM. Normal, *n* = 7; CIA, *n* = 6; CIA+PEMF 10 Hz, *n* = 10. Significance was measured using a two-tailed Student’s *t*-test (* *p* < 0.05 and *** *p* < 0.001).

IL-6 and the cognate IL-6R produced after collagen type II immunization contribute to the pathogenesis of RA [29]. In addition, the TNF/TNFR pathway plays a prominent role in the pathogenesis of chronic inflammatory diseases and autoimmune disorders [30]. Therefore, we evaluated whether IL-6R and TNFR1 decreased in CIA+PEMF mice. Tissue IL-6R and TNFR1 levels in CIA mice significantly increased compared with untreated, normal mice (Figure 7). In the CIA+PEMF group, tissue IL-6R-positive and TNFR1-positive cells significantly reduced compared with the CIA group (Figure 7). These results suggest that PEMF reduces the responsiveness to IL-6 and TNF-α.

## 3. Discussion

RA is the most common form of inflammatory arthritis which results in a substantial social burden in terms of medical cost and loss of productivity [31]. Delay in treatment after the onset of disease symptoms is associated with worse disease outcomes, including a lower probability of disease remission after the administration of disease-modifying antirheumatic drugs (DMARD) [32]. The treatment of RA has changed in the last 20 years due to the improved understanding of disease etiology and the development of new non-biological and biological antirheumatic drugs [33]. DMARDs, including traditional synthetic drugs and biological DMARDs, have received much attention over recent decades because they effectively slow disease progression and significantly reduce joint deformity. Several biological DMARDs include TNF-inhibitors (Amjevita, Renflexis, Erelzi, Cyltezo, and Imradl), anti-CD20 antibodies (Truxima and Rixathon), IL-6 receptor antibody (Kevzara), RANKL antibody (Pralia), and JAK inhibitor (Olumiant) [34]. Biologic therapies that inhibit cytokine-driven joint inflammation and damage have increased the likelihood of remission. Currently, biological therapies that target proinflammatory cytokines, such as TNF, IL-1β, or IL-6, have greatly improved the treatment of RA. However, the limitations of existing treatments create many adverse drug reactions, such as cardiovascular and gastrointestinal side effects and hypersensitivity; therefore, not all RA patients respond to current biologic therapies, and their responses are not always maintained [35]. A promising novel strategy for RA treatment is the local or systemic application of PEMF to modulate immune responses and repair tissue.

PEMF stimulates biological systems through the generation of electromagnetic fields that modify inherent electromagnetic frequencies that are generated by living organisms [13,36]. PEMF activates multiple cell types, including osteoblasts, chondrocytes, synoviocytes, and immune cells [37]. It has been reported that PEMF stimulation can inhibit NF-κB activation and regulate the synthesis and activation of proinflammatory cytokines, including TNF-α, IL-1β, and other mediators involved in joint inflammation and bone disease [15,38]. For optimal PEMF treatment, three important parameters need to be considered: frequency, intensity, and duration of exposure [13]. In a study of PEMF treatment on experimental arthritis in rats, modulation of these three parameters affected the degree of beneficial results [39]. Studies of human RA patients, which were based on a questionnaire, reported that 30 mins of treatment with PEMF can reduce arthritic pain [16]. However, short treatment most likely affected the nervous system rather than decreasing inflammation. In another report, the beneficial effects of PEMF were reported to last for several months in human patients with chronic inflammatory and autoimmune disorders without obvious indications of adverse effects [40]. PEMF is showing promise as a treatment for autoimmune diseases by modulating proinflammatory cytokine expression and cell signaling pathways to restore them to normal homeostatic levels [41].

To our knowledge, the current study is the first demonstration that PEMF can ameliorate CIA in a mouse model. The clinical arthritic score and histological evidence clearly showed that PEMF ameliorated hind paw deterioration compared with the untreated CIA group. Specifically, bone, cartilage, and synovium deterioration decreased, and fewer activated osteoclasts were evident based on the reduction of TRAP-positive osteoclasts in the bone erosion region. These findings suggest that the functional damage of chronically inflamed joints and the structural and bone erosion of cartilage are improved. Two different PEMF frequencies (10 Hz and 75 Hz) both showed a statistically significant improvement in arthritic scores compared with the CIA group. All subsequent examinations were performed on the 10 Hz PEMF treatment group because 10 Hz PEMF was slightly more effective than 75 Hz PEMF treatment. In our study, only two boundary values of 10 Hz and 75 Hz were used; therefore, the relationship between frequency and treatment efficacy cannot be determined. It is possible that other frequencies may be more effective, which needs to be examined further. In the current study, PEMF exposure time was continuous, which is not realistic for the application of PEMF treatment in human RA patients. For human application, a more realistic scenario is PEMF treatment for several hours per day. Along these lines, the frequency may need to be re-examined for human application as different frequencies, other than the ones used in the current study, may be more effective in human RA patients. We are now in a position to conduct mechanistic studies of PEMF in the CIA model.

Proinflammatory cytokines play a pivotal role in RA. Synovial macrophages can produce inflammatory cytokines, such as IL-1β, IL-6, and TNF-α, but they also indirectly stimulate osteoclast production by inducing RANKL expression in osteoblasts and synovial fibroblasts [42]. It has also been reported that RANKL is induced by IL-6 signaling and stimulates IL-6 production in synovial fibroblasts by IL-17, IL-1β, and TNF-α [43]. In the current study, as expected, the serum levels of IL-1β, IL-6, IL-17, and TNF-α in CIA mice significantly elevated compared with the serum levels in normal mice. Of the four serum proinflammatory cytokines examined (IL-1β, IL-6, IL-17, and TNF-α), only IL-1β levels statistically significantly decreased in the CIA+PEMF group compared with the CIA group (Figure 3). However, serum levels of IL-6 (*p* = 0.15) and IL-17 (*p* = 0.11) in the CIA+PEMF group did show a minor decrease compared with the CIA group, but they did not reach statistical significance. Thus, the discrepancy between arthritis scores and serum cytokines levels in the CIA+PEMF group and the CIA group is difficult to reconcile. The ameliorative effect of PEMF is likely not due to the decrease in serum IL-1β alone. It is possible that PEMF increased the secretion of anti-inflammatory cytokines, which was not examined in our study, unfortunately.

We found a marked reduction in IL-1β, IL-6, and TNF-α during immunohistochemical examination of tissue cytokine levels. In addition to increased tissue cytokine levels, the presence of corresponding cytokine receptors to IL-6 (IL-6R) and TNF-α (TNFR1) also decreased. These results strongly indicate that the evaluation of tissue cytokine and cytokine receptor levels is more informative of disease progression than serum analysis.

In the murine CIA model, anti-collagen type II IgG titers correlated with arthritic dis-ease severity [44]. Although a modest but statistically insignificant increase in anti-collagen type II IgG2b titer was detected, we found no evidence that PEMF attenuated anti-collagen-specific antibody levels. We speculate that this result may be due to several possibilities. The first possibility is that PEMF exerted no beneficial effect on antibody formation due to its ineffectiveness in down-regulating TNF-α. Kruglov et al. showed that TNF controls autoantibody formation in the CIA model [45]. Since PEMF treatment did not affect serum TNF-α levels, the production of autoantibodies may have remained unchanged. The second possibility is that the correlation between anti-collagen-specific antibody titer and arthritic severity only applies during the initial stages of RA progression. Williams et al. examined anti-collagen autoantibody titers and arthritic severity using the same mouse model for CIA that our study used. They found a high correlation between anti-collagen antibody titers and arthritic severity before the actual development of arthritis. In another study, no correlation was found between autoantibody levels and arthritic scores [46]. In our current study, PEMF treatment started when the mice began showing symptoms of arthritis, and the antibody titer levels were examined at the termination of the experiment (i.e., 60 days post-initial injection). Considering that the half-life of murine IgG is approximately 21 days, we have detected autoantibodies that formed before PEMF treatment. The third, more intriguing possibility is that PEMF affected the glycosylation of autoantibodies. It has been proposed that the glycosylation of antibodies to citrullinated proteins plays an important role in RA pathogenesis (reviewed in [47]). Anti-citrullinated antibodies which were hyposialyated and/or hypogalactosylated were pathogenic; however, adequately glycosylated anti-citrullinated antibodies were non-pathogenic. In future studies, periodic examination of autoantibody titers from the beginning of PEMF treatment as well as the glycosylation status of autoantibodies may be more informative for understanding the effects of PEMF in the CIA model.

In addition to the high serum autoantibody titers, high serum proinflammatory cytokine levels (with the exception of IL-1β) were maintained in the CIA+PEMF group. Serum cytokines have a much shorter half-life compared with serum antibodies, and we expected a decrease in serum cytokine levels. It may be possible that tissues other than the hind paws examined in this study are a source of these cytokines. Hansson et al. examined extra-articular damage in the rat CIA model [48]. They found evidence of tissue damage in nasal and tracheolaryngeal cartilage, which suggests that although joint inflammation is the main focus of numerous murine CIA studies, other tissues are affected as well. In our study, PEMF was not localized to the hind paws; therefore, the ameliorative effects of PEMF treatment should be systematic. However, we cannot rule out the possibility that PEMF did not exert an effect on other tissues. Treatment begins at the time of collagen injection in many studies that use the CIA model to evaluate therapeutic effectiveness. In our study, PEMF treatment was initiated at disease onset. This difference is meaningful because RA patients will undergo diagnosis and treatment only after disease symptoms are evident. Thus, the current study is more reflective of a real-life situation.

In a previous study, we demonstrated the protective effects of PEMF treatment on septic shock mortality in an LPS-induced murine sepsis model [49]. This study showed that PEMF can also ameliorate both chronic autoimmune disease and acute inflammatory responses. These two different model systems involve proinflammatory cytokine production. As a consequence, the hypothesis that PEMF exerts therapeutic effects through the modulation of inflammatory cytokines is compelling. There is still no general agreement regarding the exact mechanism elicited by PEMF on biological systems. Compounding this problem, the biological effects of PEMF appear to be dependent on frequency, amplitude, timing, and length of exposure, as well as the intrinsic susceptibility and responsiveness of different cell types. Future research will need to focus on the effect of PEMF on cells to identify the cellular signaling pathways that are involved and to pinpoint the mechanistic role of PEMF on these pathways.

In summary, we found that PEMF treatment ameliorated RA in the CIA model. Tissue cytokine levels and the corresponding cytokine receptor levels showed a correlation with arthritic severity.

## 4. Materials and Methods

### 4.1. Animal Studies

Male DBA/1 mice (6–7 weeks old) were purchased from Orient Bio (Seongnam, Republic of Korea) and separated into 4 groups: Normal, *n* = 12; CIA, *n* = 16; CIA+PEMF 10 Hz, *n* = 24; CIA+PEMF 75 Hz, *n* = 12. Mice were maintained in individually ventilated cages in an air-conditioned room at 23 ± 1 °C with a 12 h light/12 h dark cycle and had access ad libitum to food and water. Mice were acclimated for 1 week after purchase, and they were then used for experiments. Bovine type II collagen was used to induce arthritis in mice, as described previously [19]. Briefly, bovine type II collagen (2 mg/mL) was mixed at a 1:1 volume ratio with complete Freund’s adjuvant (CFA, Chondrex, Seattle, WA, USA). Mice were anesthetized with isoflurane, and a 26-gauge needle was used to subcutaneously inject 100 µg of bovine type II collagen in 0.1 mL of emulsion into the tail 1.5 cm distal from the base. Injection into adjacent blood vessels was avoided. A booster injection of 100 µg of bovine type II collagen was administered subcutaneously as a solution in 0.1 mL of incomplete Freund’s adjuvant (IFA, Chondrex) 21 days later. PEMF treatment was performed by exposing animals continuously starting on day 28 (Supplementary Figure S1). The PEMF device has six coils (6 cm in diameter in a circular form) and emits a magnetic field at a frequency between 1 and 2000 Hz in fountain form. The intensity of the magnetic field was measured using a gauss meter (Lake Shore Cryotronics, Westerville, OH, USA), and the average value was obtained (Supplementary Table S1). Animals were placed in mouse cages 1 cm above the PEMF generator (Supplementary Figure S2). PEMF treatment for mice was performed at room temperature for 30 consecutive days. Experimental protocols were approved by the Institutional Animal Care and Use Committee (IACUC) of Yonsei University MIRAE Campus in accordance with the regulations of the Association for the Assessment and Accreditation of Laboratory Animal Care International (#YWCL-201904-005-03).

### 4.2. Clinical Assessment of CIA

Each mouse was clinically evaluated twice weekly. A blinded observer scored each paw on a scale of 0–4. The scale was as follows: 0, normal; 1, swelling of one or more digits; 2, erythema and mild swelling of the ankle joint; 3, erythema and moderate swelling involving the entire paw; and 4, severe erythema and swelling of the limbs or ankylosis. The average macroscopic score was expressed as a cumulative value for all paws with a maximum possible score of 16 (Supplementary Figure S3).

### 4.3. Assessment of Arthritis and Histologic Score

The hind paws were fixed with 10% neutral-buffered formalin (Dana Korea, Incheon, Republic of Korea) for 3 days and decalcified in 5% formic acid (Duksan, Ansan, Republic of Korea) for 3 days at room temperature. The decalcified paws were neutralized in 0.25% lithium carbonate (Sigma-Aldrich, St. Louis, MO, USA) for 1 h and subjected to routine histological processing. Formalin-fixed paraffin-embedded (FFPE) blocks were sectioned at 4 μm using a microtome RM2125 (Leica, Wetzlar, Germany). Sagittal serial sections of the hind paws were cut and stained with hematoxylin (YD Diagnostics, Yongin, Republic of Korea) and eosin (BBC Biochemical, Stanwood, WA, USA) or safranin O (Sigma-Aldrich) for light microscopy examination. Images were examined by light microscopy (Leica, Wetzlar, Germany) and rendered using Leica software. Sections were analyzed microscopically for the degree of inflammation and cartilage and bone destruction according to previously published methods using the following scale: 0, no inflammation; 1, mild focal infiltration; 2, moderate infiltration; 3, severe infiltration without cartilage damage nor pannus formation; and 4, extremely serious inflammatory infiltration, pannus formation, and/or cartilage damage (Supplementary Figure S4). The following scale was also used: 0, normal synovium; 1, decreased safranin O staining without structural changes; 2, vertical clefts/erosion to the calcified cartilage extending to <30% of the articular surface; 3, vertical clefts/erosion to the calcified cartilage extending between 30–60% of the articular surface; and 4, vertical clefts/erosion to the calcified cartilage extending to >60% of the articular surface (Supplementary Figure S5). The average macroscopic score was expressed as a cumulative value for hind paws with a maximum possible score of 8.

### 4.4. Immunohistopathological Analyses of Arthritis

Ankle joint tissue sections were attached to silane-coated slides (Muto Pure Chemicals Co., Ltd., Tokyo, Japan), baked, and de-paraffinized. Antigen retrieval was performed in a citrate buffer (pH 6.0) overnight at 60 °C. Sections were blocked for 30 min with 1.5% bovine serum albumin (Sigma, St. Louis, MO, USA), followed by incubation with primary antibody against IL-6 (R&D Systems, Minneapolis, CA, USA), IL-1β (R&D Systems), TNF-α (R&D Systems), RANK (R&D Systems), RANKL (R&D Systems), IL-6R (Invitrogen, Waltham, MA, USA), or TNF-αR (Invitrogen) overnight at 4 °C (Supplementary Table S2). Biotinylated secondary antibodies (Jackson ImmunoResearch, West Grove, PA, USA) were used to detect primary antibodies. Thereafter, a streptavidin-tagged horseradish peroxidase kit (Vector Laboratories, Burlingame, CA, USA) was used to amplify the signal, and visualization of the antibody was performed using a NovaRED detection kit (Vector Laboratories, Burlingame, CA, USA). Mayer’s hematoxylin (BBC Biochemical, Stanwood, WA, USA) was used as a counterstain. Stained slides were photographed by optical microscopy (Leica, Wetzlar, Germany) and rendered using Leica software. A light microscope was used for image processing, and immunohistochemistry signals were quantified using ImageJ software (Version 1.53, NIH, Bethesda, MD, USA).

### 4.5. Tartrate-Resistant Acid Phosphatase (TRAP) Staining

Decalcified FFPE serial sections were stained using a TRACP & ALP double staining kit (Takara, Shiga, Japan) for 1 h at 37 °C. The standard naphthol AS-BI phosphate postcoupling method was used for each section, and Fast Red Violet LB was used as the coupler where naphthol phosphate cleavage gave rise to a red precipitate of a diazonium product. The reaction was performed by adding the substrate solution for acid phosphatase (NABP/FRVLB) which was supplemented with tartrate. Samples that were not supplemented with tartrate served as a negative control. TRAP-positive osteoclasts were visualized using a light microscope (Leica).

### 4.6. Enzyme-Linked Immunosorbent Assay (ELISA)

Blood was harvested from mice at the end of the experiment, and serum was prepared by centrifugation at 2000 g for 15 min. The serum was stored at −80 °C until analysis. Serum cytokine levels (IL-6, IL-1β, IL-17, and TNF-α; R&D Systems) and autoantibodies (anti-type II collagen IgG, IgG1, IgG2a, and IgG2b; Chondrex, Seattle, WA, USA) were measured with commercially available ELISA kits according to the manufacturer’s instructions. The intensity of the color was measured at 450 nm using an In-finite M200 PRO Multimode Microplate Reader (TECAN U.S. Inc., Research Triangle Park, NC, USA).

### 4.7. Statistical Analysis

Comparison of the median was performed using the unpaired two-tailed Student’s *t*-test unless otherwise indicated. Statistical analyses were evaluated using GraphPad Prism 5.0 (GraphPad Software Inc., CA, USA). A probability value (*p*) less than 0.05 (*p* < 0.05) indicated a statistically significant difference.

## Data Availability

The data presented in this study are available upon request from the corresponding author (K.-J.R).

## Supplementary Materials

The following supporting information can be downloaded at: https://www.mdpi.com/article/10.3390/ijms24021137/s1, Figure S1: PEMF treatment scheme; Figure S2: PEMF device; Figure S3: Clinicopathologic severity scoring of arthritis in CIA mice; Figure S4: Histologic inflammation scoring in CIA mice; Figure S5: Cartilage damage scoring in CIA mice; Table S1: PEMF intensity relative to coil distance; Table S2: Antibodies used in this study.
