# Supplementary material for: Pulsed Electromagnetic Field (PEMF) Treatment Ameliorates Murine Model of Collagen-Induced Arthritis
Source: Int J Mol Sci. 2023 Jan 6;24(2):1137. doi: 10.3390/ijms24021137 (PMC9862561; doi:10.3390/ijms24021137)
Supplement: Supplementary file 1 [file ijms-24-01137-s001.zip › ijms-2090812-supplementary.pdf]

[Supplementary data to International Journal of Molecular Sciences]

Pulsed Electromagnetic Field (PEMF) Treatment Ameliorates Murine Model of Collagen-Induced Arthritis

Ju-Eun Hong [1], Chang-Gun Lee [1,2], Soonjae Hwang [1,3], Junyoung Kim [4], Minjeong Jo [1,5], Da-Hye Kang [1], Sang-Hyeon Yoo [1], Woo-Seung Kim [1], Yongheum Lee [4] and Ki-Jong Rhee [1,*]

[1] Department of Biomedical Laboratory Science, College of Software and Digital Healthcare Convergence, Yonsei University MIRAE Campus, Wonju 26493, Republic of Korea

[2] Department of Medical Genetics, Ajou University School of Medicine, Suwon 16499, Republic of Korea

[3] Department of Biochemistry, Lee Gil Ya Cancer and Diabetes Institute, GAIHST, Gachon University College of Medicine, Incheon 21999, Republic of Korea

[4] Department of Biomedical Engineering, College of Software and Digital Healthcare Convergence, Yonsei University MIRAE Campus, Wonju 26493, Republic of Korea

[5] Department of Molecular Cell Biology, Sungkyunkwan University School of Medicine, Suwon 16419, Republic of Korea

* Correspondence: kjrhee@yonsei.ac.kr; Tel.: +82-33-760-2445; Fax: +82-33-760-2195

Supplementary Figure S1. PEMF treatment scheme

Supplementary Figure S2. PEMF device

Supplementary Figure S3. Clinicopathologic severity scoring of arthritis in CIA mice

Supplementary Figure S4. Histologic inflammation scoring in CIA mice

Supplementary Figure S5. Cartilage damage scoring in CIA mice

Supplementary Table S1. PEMF intensity relative to coil distance

Supplementary Table S2. Antibodies used in this study

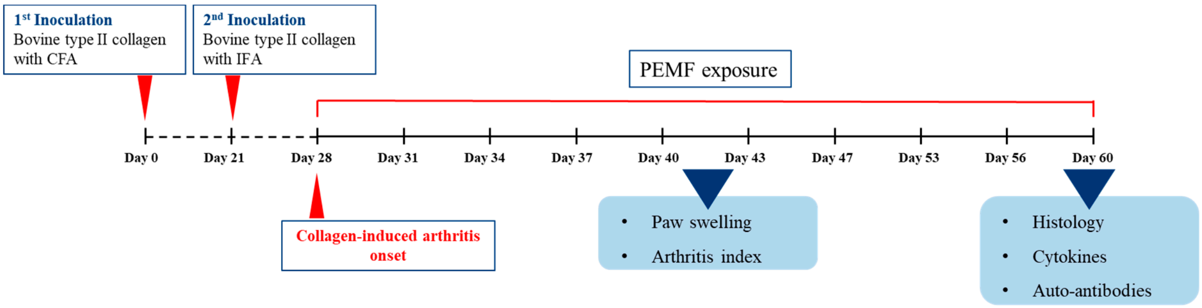

Supplementary Figure S1. PEMF treatment scheme

Mice were inoculated with bovine type II collagen and complete Freund's adjuvant (CFA) at day 0. At day 21, mice were inoculated with bovine type II collagen and incomplete Freund's adjuvant (IFA). At day 28, mice were continuosly treated with PEMF (10 Hz or 75 Hz) until termination of the experiment (day 60). Clinicopathologic evaluation (paw swelling, arthritis index) was performed from day 28 every 2-3 days. At day 60, sera and tissues were harvested and examined.

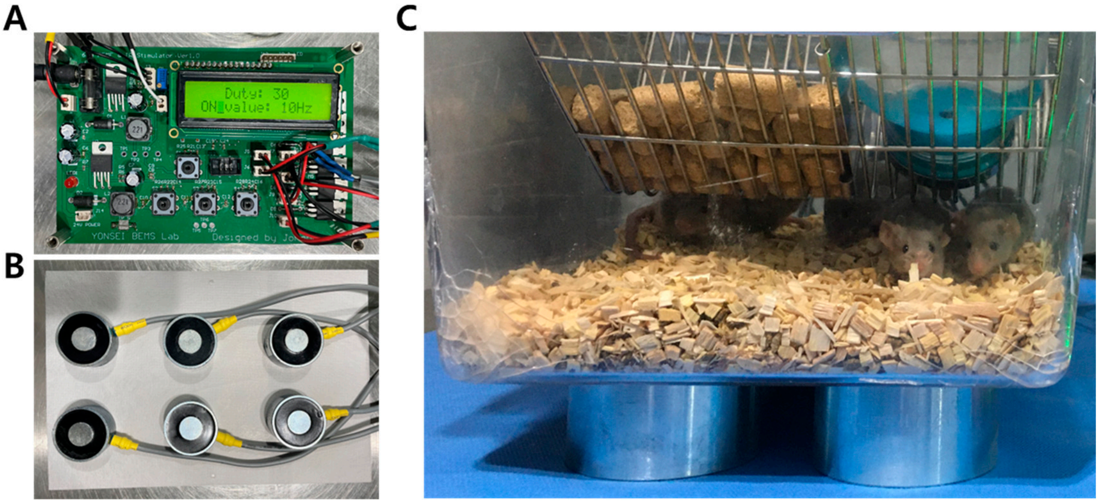

Supplementary Figure S2. PEMF device

(A) PEMF control panel. The control panel allows regulation of Hz. (B) PEMF generator. Six PEMF coils were placed under the mouse cage. (C) Placement of mouse cages on PEMF coils. The set of six cores were placed underneath the mouse housing cage and the mice were exposed to PEMF continuously.

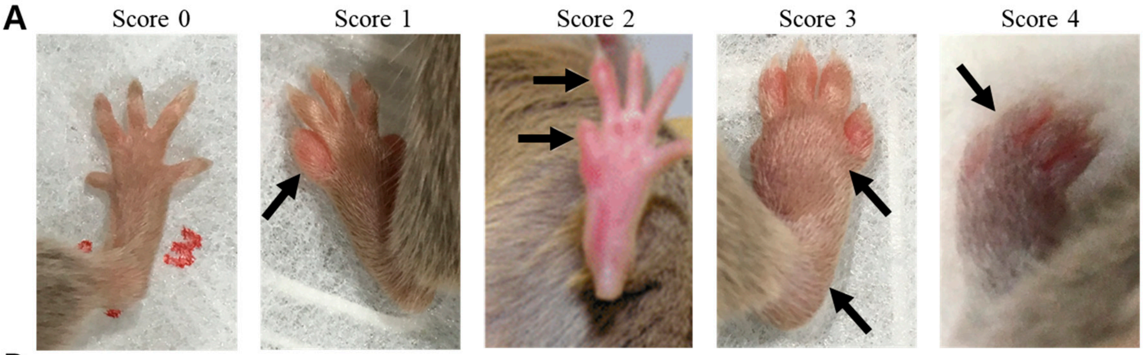

B

Score 0	Normal
Score 1	Swelling of one or more digits
Score 2	Erythema and mild swelling of the ankle joint
Score 3	Erythema and moderate swelling involving the entire paw
Score 4	Severe erythema and swelling of the limbs or ankylosis

Supplementary Figure S3. Clinicopathologic severity scoring of arthritis in CIA mice
(A) Gross images of mouse paws after CIA induction. Representative images are shown. (B) Scoring scheme for assessing arthritis severity.

A

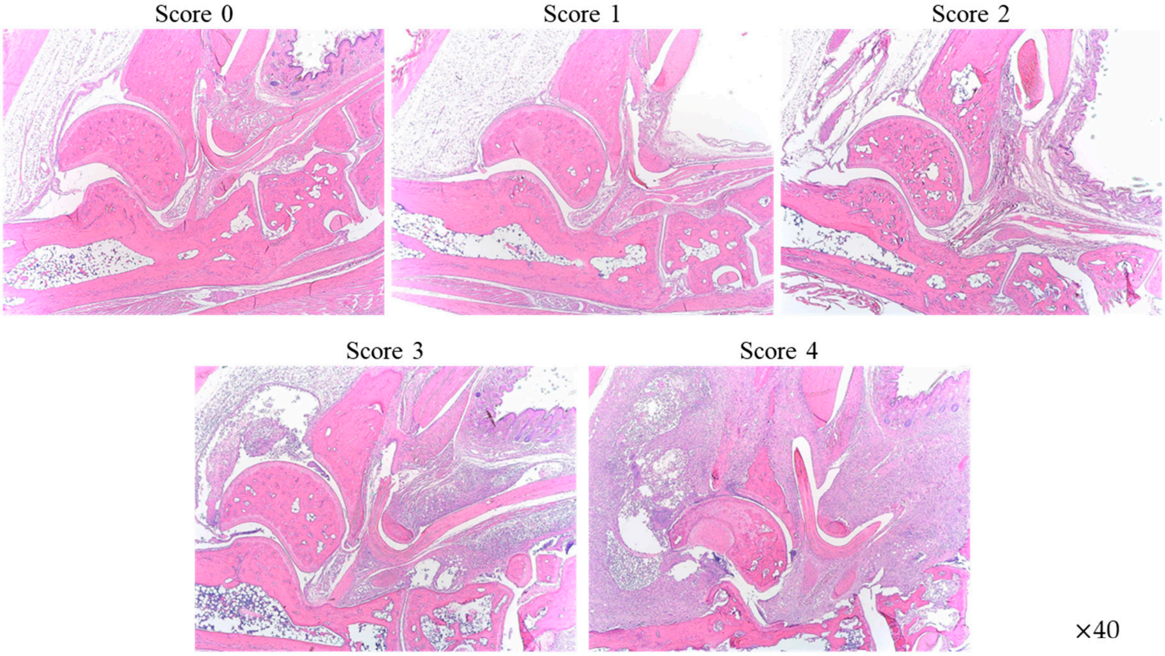

B

Score 0	No inflammation
Score 1	Mild focal infiltration
Score 2	Moderate infiltration
Score 3	Severe infiltration without cartilage damage nor pannus formation
Score 4	Extremely serious inflammatory infiltration, pannus formation and/or cartilage damage

Supplementary Figure S4. Histologic inflammation scoring in CIA mice

(A) H&E stained sections of mouse arthritic regions of mouse paws. Representative images are shown.

(B) Scoring scheme for assessing arthritis severity of histologic sections.

A

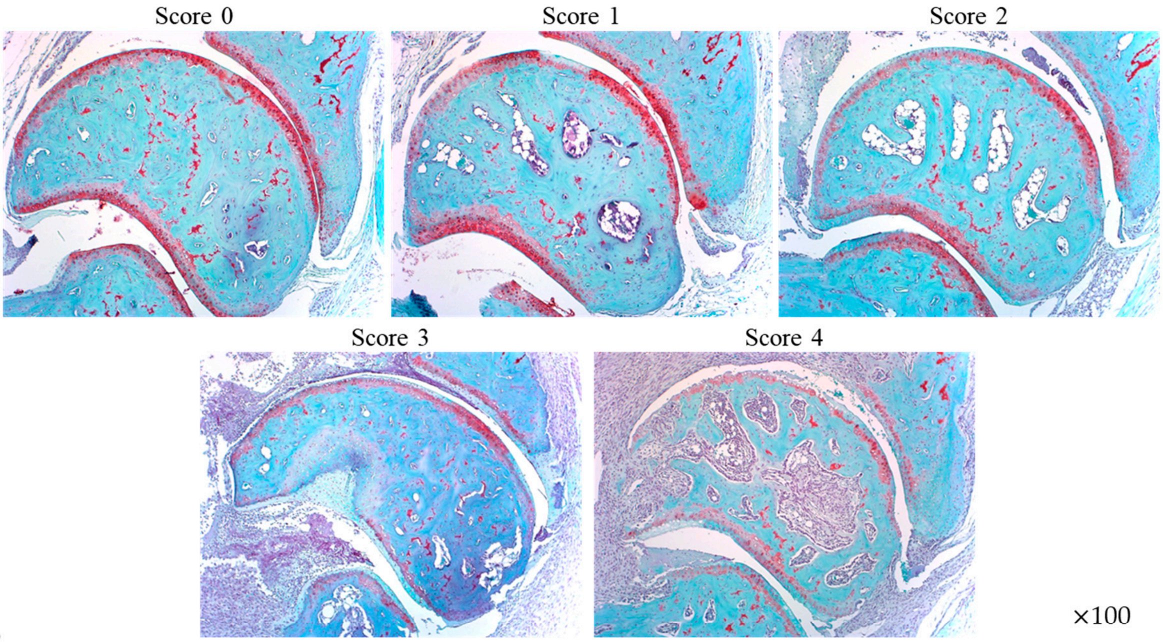

B

Score 0	Normal synovium
Score 1	Decreased safranin O staining without structural changes
Score 2	Vertical clefts/erosion to the calcified cartilage extending to <30% of the articular surface
Score 3	Vertical clefts/erosion to the calcified cartilage extending to 30-60% of the articular surface
Score 4	Vertical clefts/erosion to the calcified cartilage extending to >60% of the articular surface

Supplementary Figure S5. Cartilage damage scoring in CIA mice

(A) Safranin O stained sections of mouse arthritic regions of mouse paws. Representative images are shown. (B) Scoring scheme for assessing cartilage damage.

Supplementary Table S1. PEMF intensity relative to coil distance

Distance (cm)	Hertz (Hz)	Gauss (G)
0~1	10	262
	75	146.7
1~2	10	145
	75	85.9
2~3	10	46
	75	45.2

The intensity (Gauss) differs based on the Hertz setting and also the distance from the magnetic coil. The intensity of PEMF from the distance from the coil.

Supplementary Table S2. Antibodies used in this study

Target protein	Concentration/ Dilution Factor	Company (Cat. No.)
IL-6	3 ug/ml	R&D Systems (AF-406)
IL-1β	3 ug/ml	R&D Systems (AF-401)
TNF-α	2 ug/ml	R&D Systems (AF-410)
RANK	3 ug/ml	R&D Systems (AF-692)
RANKL	3 ug/ml	R&D Systems (AF-462)
IL-6R	1:300	Invitrogen (PA5-102425)
TNFR1	1:300	Invitrogen (33-0100)
Rabbit anti-Goat IgG (H&L)	1:500	Jackson ImmunoResearch (305-065-003)
Goat Anti-Mouse IgG (H&L)	1:500	Jackson ImmunoResearch (115-065-166)
Goat Anti-Rabbit IgG (H&L)	1:500	Jackson ImmunoResearch (111-065-003)
